# On Scarlet Fever Complicated with Rheumatic and Cardiac Affections

**Published:** 1863-08

**Authors:** 


### ON SCARLET FEVER COMPLICATED WITH RHEUMATIC AND CARDIAC AFFECTIONS.

[It seems now pretty well decided that cases of scarlet fever occur, complicated with rheumatic affections of the joints, and even with well marked pericardiac and endocardiac inflammation. Dr. Richardson, in his Clinical Essays, article "Scarlet Fever," p. 85, says:]

That he once attended four children in one family for scarlet fever, and that in two of the cases well-marked symptoms of rheumatic fever set in on the second day of the eruption. In one, the endocardial membrane became affected. He writes:—"I could not make out satisfactorily any proof of hereditary taint, as accounting for the rheumatic complication; but there it was, and there was the fact, in spite of any hypothesis to the contrary, that two diseases may exist in the same body at the same time."

[The connexion of affections of the pericardium and endocardium with scarlet fever has been long recognized, but does not seem to be considered as a frequent cause of disease of the heart. It is not mentioned by such standard authors as Walshe and Markham. Dr. West, in his work on "Diseases of Children," writes:]

"In two cases of pericarditis, in three of acute and one of chronic endocarditis, or in six out of thirty-nine instances, the disease of the heart was traced to an attack of scarlet fever. The cardiac symptoms did not manifest themselves in the acute stage of the affection, but during the progress of desquamation."

In reference to the connexion of the heart affection in scarlet fever, when complicated with rheumatism, the following quotation from Dr. Watson will be interesting:—"I have several times, when the rash of scarlet fever was disappearing, known pain and swelling of the larger joints to supervene, simulating closely the local phenomena of sub-acute rheumatism; and I have noticed that the painful joints were eased and benefited by friction,—a circumstance which may help to distinguish this articular affection from true rhuematism. Another distinctive circumstance seemed to be that, although all these patients were children, the heart in no instance became implicated in connexion with the tumid joints. Upon this point, however, my own experience may have been fallacious. Dr. Scott Alison has recently invited attention to the subjeet, in an interesting essay on 'Pericarditis a Complication and Sequela of Scarlatina.' Accepting his facts, I should ascribe the articular affection and the cardiac affection, whether they occurred together or separately, to one and the same cause, viz.: to the retention in the blood of a poisonous excrement, by the default of the principal emunctories, and especially the kidney."

[In one case which occurred to Dr. Budd, at King's College Hospital, the report of the autopsy relates that there was "some recent lymph on the surface of the heart, and a few ounces of serous fluid in the pericardium." In this case no abnormal sound was heard before death, though the action of the heart was irregular and feeble. We do not consider this a common complication of scarlet fever, yet that it does occur we ourselves can testify.]—*Medical Times and Gazette.*

